# *Akkermansia muciniphila* alleviates cognitive impairment and neuroinflammation induced by blunt chest trauma

**DOI:** 10.3389/fimmu.2025.1657524

**Published:** 2025-10-01

**Authors:** Lin Liu, Linxiao Wang, Yanjun Wang, Heping Zhao, Xi Gao, Wen Yin, Jiangang Xie

**Affiliations:** ^1^ Department of Emergency, Honghui Hospital, School of Medicine, Xi’an Jiao Tong University, Shaanxi, Xi’an, China; ^2^ Department of Emergency, First Affiliated Hospital of Air Force Medical University, Shaanxi, Xi’an, China; ^3^ Department of Clinical Laboratory, Honghui Hospital, School of Medicine, Xi’an Jiao Tong University, Shaanxi, Xi’an, China; ^4^ Department of Interventional Vascular, Xi’an No.3 Hospital, Affiliated Hospital of Northwest University, Shaanxi, Xi’an, China; ^5^ Xi’an Key Laboratory of Metabolic Disease Imaging, Xi’an No.3 Hospital, Affiliated Hospital of Northwest University, Xi’an, China

**Keywords:** Akkermansia muciniphila, blunt chest trauma, cognitive function, neuroinflammation, microglia, BDNF

## Abstract

**Background:**

Blunt chest trauma, commonly caused by traffic accidents, falls, and violent incidents, results in both direct mechanical injury to the thoracic cavity—leading to increased intrathoracic pressure and vascular rupture—and indirect effects on the central nervous system (CNS), causing extensive damage that severely impacts patient health and quality of life. Akkermansia muciniphila (AKK), a probiotic bacterium inhabiting the gut mucus layer, modulates gut microbiota and metabolites, with potential therapeutic effects on various neurological disorders through the gut-brain axis.

**Methods:**

Mice were divided into four groups: control, trauma, trauma+PBS, and trauma+AKK. AKK bacterial suspension was administered *via* gavage for three weeks. Behavioral tests including the OFT, EPM, NORT, and Y-maze were conducted to assess anxiety-like behaviors and cognitive function. Neuroinflammatory markers in the hippocampus were measured using qPCR, immunofluorescence, and Western blot. Gut microbiota and metabolites were analyzed through 16S rRNA sequencing and metabolomics.

**Results:**

Mice subjected to blunt chest trauma displayed emotional abnormalities and cognitive deficits. AKK treatment significantly alleviated anxiety-like behaviors and improved cognitive function, reduced pro-inflammatory cytokine levels in the hippocampus, and reshaped gut microbiota composition. AKK also modulated the expression of metabolites linked to neuroinflammation and cognitive function, upregulated BDNF and TrkB, and decreased IBA1, suggesting it enhances cognitive function by modulating neuroinflammation and the BDNF/TrkB signaling pathway.

**Conclusions:**

AKK mitigates cognitive impairment and neuroinflammation after blunt chest trauma by modulating gut microbiota and metabolites. Targeting the gut-brain axis may offer new strategies for preventing and treating trauma-induced neurological disorders.

## Introduction

Unintentional injuries and accidents rank as the leading cause of death in individuals under 45 years of age and the third leading cause of mortality overall (1). Approximately 25% of trauma-related deaths are attributed to chest injuries or their complications (2), with blunt chest trauma comprising 70% of such cases (3, 4). This form of trauma can cause a rapid rise in intrathoracic pressure, rupture of critical blood vessels such as the thoracic aorta, and life-threatening conditions like airway obstruction, pneumothorax, and hemothorax (5). Furthermore, hemodynamic changes resulting from blunt chest trauma can impair cerebral blood flow, leading to brain tissue hypoxia (6). Chest injuries also activate the sympathetic nervous system, triggering a systemic stress response that increases blood pressure and heart rate, exacerbating cerebral hypoxia. Simultaneously, immune activation releases inflammatory cells and cytokines into the bloodstream, which can cross the blood-brain barrier, enter the CNS, and induce neuroinflammation, causing neuronal damage (7). Approximately 50% of patients with blunt chest trauma experience persistent pain, which can lead to neuroinflammation and cognitive deficits, such as memory impairment and diminished cognitive function (8). Current treatments for acute chest trauma primarily emphasize early mobilization and respiratory support, while post-traumatic neuropathic pain remains underdiagnosed (9). Secondary neurological damage following blunt chest trauma significantly impacts long-term prognosis.

Blunt chest trauma also activates both sympathetic and parasympathetic pathways, leading to gastrointestinal dysfunction, including reduced motility, vasoconstriction, and gut microbiota dysbiosis. This imbalance can produce toxins and metabolites that activate the immune system *via* the gut-brain axis, influencing CNS function and inducing neuroinflammation and neurodegeneration, which impair cognitive function (10). Numerous studies have demonstrated that modulating the composition and abundance of gut microbiota can influence immune responses and CNS function by altering the release of neuroactive substances, metabolites, and hormones (11, 12). Li et al. showed that inhibiting LPS-induced gut microbiota alterations in mice improves cognitive dysfunction (13). Wang et al., through Mendelian randomization analysis, found that gut microbiota-targeted interventions can enhance cognitive function (14). Thus, modulating the gut-brain axis represents a promising strategy for mitigating neurological disorders following blunt chest trauma.

*Akkermansia muciniphila* (AKK) is a probiotic bacterium that resides in the outer mucus layer of the gastrointestinal tract. It plays a critical role in maintaining gut barrier integrity and regulating host metabolism, including glucose and lipid metabolism, inflammatory responses, and gut barrier function (15–17). Increasing evidence highlights a strong correlation between AKK abundance and behaviors related to emotion and learning, particularly in the context of gut microbiota reshaping (18–20). AKK has been shown to reduce inflammatory markers and regulate gut metabolites, such as short-chain fatty acids (SCFAs) and neurotransmitters, which influence cognitive function (21, 22). However, the mechanisms through which AKK modulates the microbiota-gut-brain axis and improves neurological function in trauma-induced neurological disorders remain poorly understood.

This study aims to investigate the impact of AKK supplementation on neurological function in a mouse model of blunt chest trauma while exploring the underlying mechanisms involved. Pathological changes and neuroinflammation in the hippocampus following AKK supplementation were assessed, and microbiome and metabolomics techniques were employed to identify the potential pathways through which AKK modulates neurological disorders induced by blunt chest trauma. The findings from this study offer insights into potential therapeutic targets for preventing and treating neurological disorders following blunt trauma.

## Materials and methods

### Reagents

*Akkermansia muciniphila* (AKK, ATCC BAA-835) was obtained from Mingzhou Bio (China). Reagents for RNA reverse transcription to cDNA (Cat# 11141ES10, Yeasen) and SYBR Green (Cat# 11201ES08, Yeasen) were utilized. Western blot antibodies included IBA1 (#A30311, Nature Biosciences), pTrkB (#A46276, Nature Biosciences), TrkB (#ab108319, Cell Signaling Technology), BDNF (#A50292, Nature Biosciences), ERK1/2 (#9102, Cell Signaling Technology), pERK1/2 (#9101, Cell Signaling Technology), CREB (#9197, Cell Signaling Technology), pCREB (#9198, Cell Signaling Technology), and β-actin (#4967, Cell Signaling Technology).

### Animals

Sixty male C57BL/6J mice (Charles River, Germany), weighing 23 ± 3g, were used. All mice were acclimated for 14 days prior to treatment and were age-matched (approximately 10 weeks old). The animals were housed in a controlled environment (21 ± 2°C, 50% relative humidity) with ad libitum access to food and water. Only male C57BL/6J mice were used in this preliminary study due to sex-related differences in hormonal and immune responses (23, 24). The study was approved by the Health Science Center of XJTU Approval for Research Involving Animals (approval number: XJTYAE2024-1396).

### Group distribution and experimental procedures

Mice were randomly assigned to one of four groups: Control (no treatment), Trauma, Trauma+PBS (100 µL sterile PBS daily), and Trauma+AKK (100 µL AKK bacterial suspension at 2 × 10^9^ CFU/mL daily). The AKK suspension was administered *via* gavage for three weeks.

### Blunt thoracic trauma

Blunt chest trauma was induced as previously described. Oxygen was turned on and the flow rate was adjusted to 0.25 MPa, 1 L/min. The concentration of the anesthetic (isoflurane) was set to 5%, and induction anesthesia was completed in approximately 1 minute. Subsequently, the anesthetic concentration was adjusted to 2%, and a mouse mask was connected for continuous inhalation. Mice were anesthetized with isoflurane gas, and a cylindrical weight (50 ± 2g) was dropped from a height of 300 ± 2mm onto a platform placed on the chest. The impact energy (E) was calculated using the formula E = m × g × h, where m is the weight (kg), g is the acceleration due to gravity (9.8 m/s^2^), and h is the height (m) (25, 26). Preliminary experiments indicated that a 300mm height and 50g weight (E=0.15 J) induced blunt chest trauma without causing severe complications, such as pneumothorax, hemothorax, cardiac rupture, or immediate death. Following impact, mice exhibited transient shock and rapid breathing and regained motor function after anesthesia recovery. To mitigate behavioral abnormalities caused by pain in surviving mice post-trauma, we administered meloxicam *via* subcutaneous injection at a dose of 5 mg/kg, with daily dosing for 3 days post-surgery. Additionally, we allowed sufficient time for the mice to recover from the acute pain phase post-surgery (27). To assess the consistency of pain in the mice, we monitored their pain behavior by observing the distance traveled in the open field test. There was no significant difference in the total distance traveled in the open field between trauma-induced mice and normal mice, after which we further assessed the emotional behavior of the mice.

### Open field test

Mice were acclimated to the testing environment for 1 hour prior to behavioral testing to minimize external interference. The testing arena (50cm × 50cm × 40cm) was cleaned with 75% ethanol between each test to remove residual odors. Mice were placed in the center of the arena and allowed to explore for 10 minutes. Behavioral recordings included trajectory, distance traveled, time spent, and speed, which were analyzed using Noldus EthoVision software. Reduced inner area activity and shorter exploration times were indicative of anxiety-like behavior (28).

### Elevated plus-maze test

Mice were acclimated to the testing environment for at least 1 hour prior to the test. The apparatus was cleaned between each test to ensure a scent-free environment. Mice were placed in the center of the maze facing an open arm and allowed to explore for 5 minutes. Behavior was recorded, and trajectories, distance traveled, entries into the open arms, and time spent in the open arms were analyzed using Noldus EthoVision software. Reduced activity in the open arms, indicated by shorter distances and times, was interpreted as anxiety-like behavior (29).

### Tail suspension test

The posterior third of the mouse tail was suspended from a support, with the head positioned 15cm above the table. Behavior was recorded for 5 minutes, and immobility time was analyzed using Smart v3.0 software. Increased immobility time suggested depression-like behavior (30).

### Forced swimming test

Mice were placed in a transparent cylinder (10cm diameter × 18cm height) filled with water (2/3 volume at 25 °C) for 6 minutes. Behavior was recorded during the final 5 minutes, and immobility time was analyzed using the software. Extended immobility times were indicative of depression-like behavior (30).

### Novel object recognition test

The test consisted of three phases: habituation, training, and testing. During the habituation phase, mice explored an open field for 10 minutes on the first day. On the training day, mice were exposed to two identical objects for 10 minutes. In the testing phase, mice were presented with a familiar object (A1) and a novel object (A2) for 10 minutes. Exploration was defined as touching or directing the head within 2cm of the object. All objects and the arena were cleaned with 75% ethanol between tests. The preference index was calculated as the time spent exploring the novel object (T_A2_) divided by the total exploration time for both objects (T_A1_ + T_A2_). Normal cognitive function was indicated by a preference for the novel object (29).

### Y-maze

The Y-maze test also involved three phases: habituation, training, and testing. During the habituation phase, mice were food-deprived for one day but had free access to water. Food was placed in all three arms of the Y-maze, and mice were allowed to explore for 10 minutes twice a day over two days. During training, mice were food-deprived for one day, with food placed in one arm (the food arm) and the other two arms left empty. Mice explored for 10 minutes twice a day for two to three days. In the test phase, mice were food-deprived for one day, and all three arms were empty. Mice were placed in the start arm (the non-food arm), and the number of entries and time spent in the food arm were recorded. Time spent in the food arm indicated learning and memory ability, with longer times suggesting better memory. Longer times spent in the error arm (other than the start or food arms) indicated poorer memory (31).

### Pathological staining

Hematoxylin-Eosin (HE) Staining: Tissue was fixed at room temperature for 24 hours, dehydrated, embedded in paraffin, and sectioned at 4 µm thickness. Sections were stained with hematoxylin and eosin and observed under a microscope.

Immunofluorescence (IF) Staining: Tissue sections were dewaxed, rehydrated, and subjected to antigen retrieval using citrate buffer. Endogenous peroxidase activity was blocked with 3% H_2_O_2_. Sections were incubated with primary antibodies overnight at 4°C, followed by incubation with secondary antibodies for 1 hour at room temperature. Sections were mounted with an anti-fade mounting medium and observed under a fluorescence microscope. Fluorescence intensity was quantified using ImageJ software.

### Gut microbiota 16S sequencing

16S rRNA gene sequencing was employed to analyze the gut microbiota of traumatized mice. The V3-V4 regions of the 16S rDNA were amplified *via* PCR, purified on a 2% agarose gel, and quantified. The fragments were sequenced on the Illumina platform following the manufacturer’s paired-end read protocol (PE250). Representative sequences for each operational taxonomic unit (OTU) were classified using the RDP Classifier software with a Bayesian algorithm at a confidence level of 0.8. Bioinformatics analysis was performed using the Omicsmart platform.

### Metabolomics sequencing

Metabolomic analysis was performed using an Agilent 1290 Infinity LC system coupled with an AB Triple TOF 6600 mass spectrometer. Chromatographic separation was achieved on an HSS T3 column. For positive electrospray ionization (ESI) mode, the mobile phase consisted of water with 0.1% formic acid (solvent A) and acetonitrile (solvent B). For negative ESI mode, water containing 0.5 mM ammonium formate (solvent A) and acetonitrile (solvent B) was used. Bioinformatics analysis was performed to identify potential metabolites.

### Quantitative real-time polymerase chain reaction

Total RNA was isolated using a Trizol reagent, and RNA concentration was measured. RNA was reverse-transcribed into cDNA according to the manufacturer’s instructions. For qPCR reactions, cDNA, gene-specific primers, and SYBR Green were mixed. Primer sequences are provided in **Supplementary Table 1**.

### Western blot

Total protein was extracted from tissues using RIPA lysis buffer, and protein concentration was measured using a BCA assay kit. Proteins were separated by SDS-PAGE and transferred to PVDF membranes. Membranes were incubated with primary antibodies and HRP-conjugated secondary antibodies. Protein bands were visualized using a ChemiDoc imaging system (Bio-Rad, Hercules, CA, USA), and band intensity was analyzed using ImageJ software.

### Statistical analysis

The study adhered to principles of randomization and blinding to ensure objectivity. Results are presented as mean ± standard error (SEM). Statistical analysis was conducted using unpaired t-tests, Welch’s t-tests, or one-way ANOVA with GraphPad Prism version 8. *P*-values less than 0.05 were considered statistically significant.

## Results

### Reproducibility and survival rate of the blunt thoracic trauma mouse model

Regarding the reproducibility of the blunt chest trauma mouse model, the survival rate of the mice was 69% (with 12 deaths and 27 survivals). Mice that died following trauma either succumbed immediately or within 24 hours post-trauma. Upon autopsy, these deceased mice exhibited substantial hemorrhaging within the thoracic cavity, perforation or rupture of the right atrium, yet no rib fractures or visible damage to abdominal organs were observed. Surviving mice displayed transient respiratory depression immediately after injury, subsequently presenting with rapid breathing, piloerection, lethargy, and reduced activity. These symptoms gradually normalized by day 3 post-trauma. We harvested tissues from mice 3 days post-trauma and observed contusions on the left and right lungs caused by the ribs. Histological examination of the heart and lung tissues of the trauma-induced mice *via* HE staining revealed extensive pulmonary hemorrhage and edema, with alveoli infiltrated by a large number of inflammatory cells and thickened alveolar septa. In contrast, normal mouse lung tissue exhibited intact alveolar structures with thin and uniform alveolar walls. As for the heart, trauma-induced mice showed unclear myocardial striations, disordered arrangement, vacuolization of myocardial cells, and blurred cell boundaries, while normal mouse heart tissue had neatly arranged myocardial fibers with clear structures (**Supplementary Figure 1**). These findings indicate that we have successfully induced blunt chest trauma in mice.

### Cognitive impairment and anxiety induced by trauma

**Figure 1:**
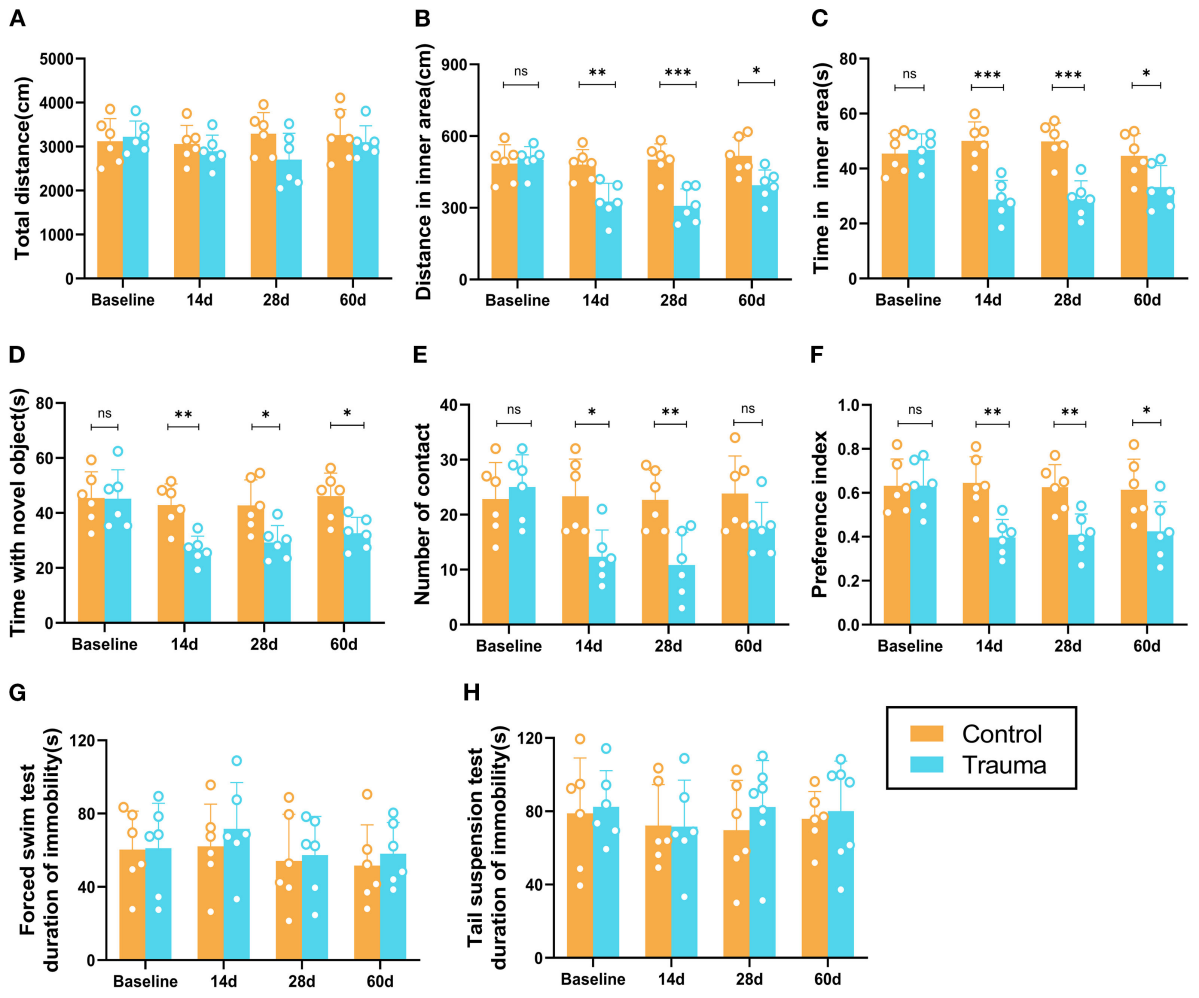
Cognitive and behavioral impairments in trauma-exposed mice. **(A-C)** Results of the OFT at different time points. **(A)** Total distance traveled by the mice in the open area. **(B)** Distance traveled in the inner area. **(C)** Time spent in the inner area. **(D-F)** Results of the novel object recognition test at various time points. **(D)** Total distance traveled by the mice. **(E)** Number of touches to the novel object. **(F)** Preference index [A2/(A1+A2)]. **(G, H)** Depression-like behaviors in the forced swimming test (FST) and tail suspension test (TST), with no significant differences observed. Data are presented as mean ± SEM. n = 6, *P < 0.05, **P<0.01, ***P< 0.001, ns, no statistical significance vs. control.

To evaluate the effects of trauma on cognitive and emotional behaviors in mice, behavioral tests were conducted to assess learning and memory, anxiety, and depression. In the OFT, the total distance traveled by traumatized mice did not significantly differ from that of controls (*P* > 0.05, **Figure 1A**), suggesting no substantial impact on overall motor function. However, from day 14 to day 60 post-trauma, traumatized mice exhibited significantly reduced distances and time spent in the inner area compared to controls (*P*<0.05, **Figures 1B, C**). In the NORT, traumatized mice showed significantly decreased exploration times and frequencies for the novel object starting at day 14 (*P*<0.05, **Figures 1D, E**), with a lower preference index (*P*<0.05, **Figure 1F**) that persisted through day 60. By day 60, however, the frequency of novel object exploration did not significantly differ from controls, indicating partial recovery of interest in novel objects. In the TST and FST, traumatized mice showed no significant differences in immobility times compared to controls (*P* > 0.05, **Figures 1G, H**). Overall, while traumatized mice exhibited anxiety and cognitive impairments from day 14 onward, no depression-like behaviors were observed.

### Gut microbiota dysbiosis induced by trauma

**Figure 2:**
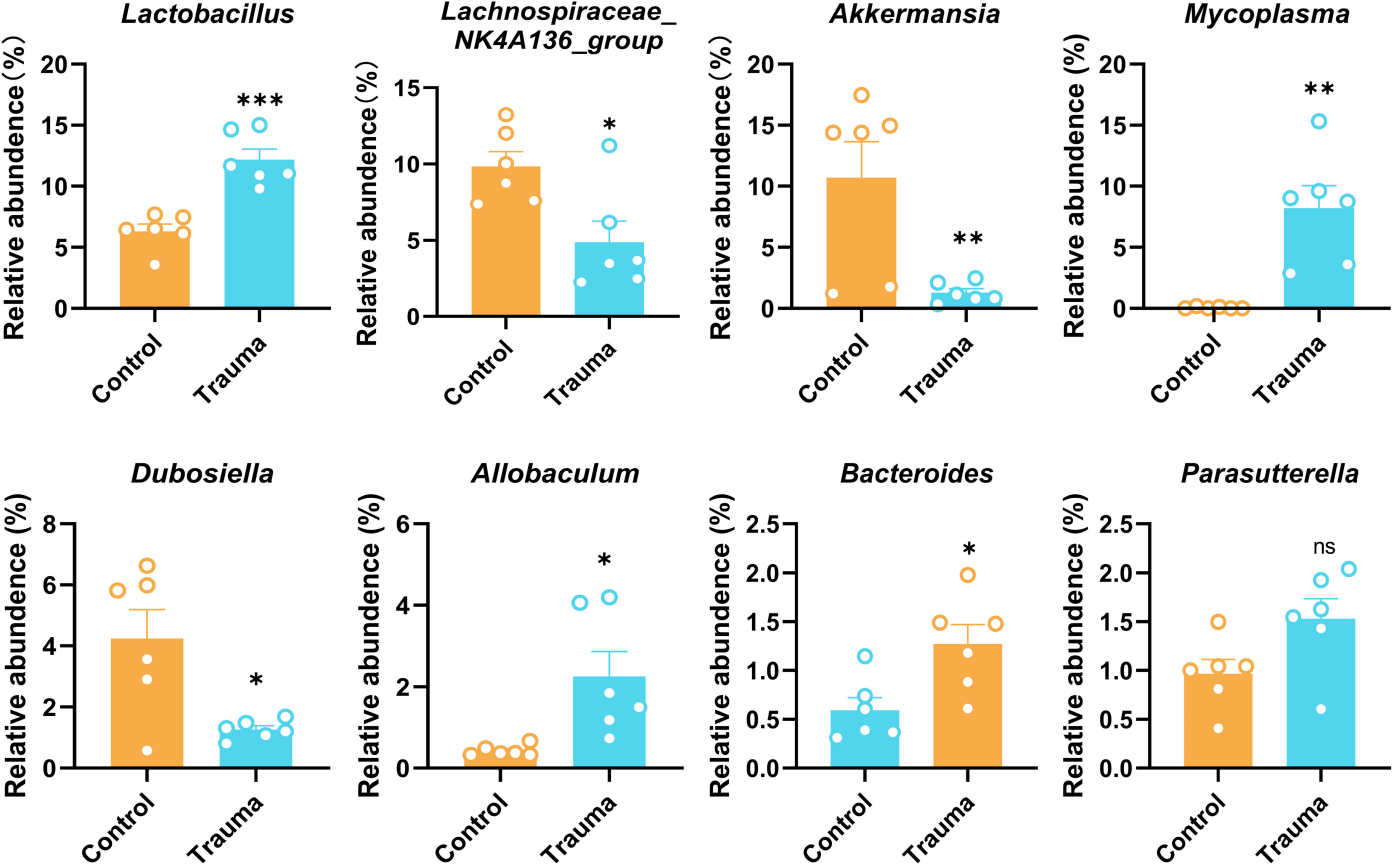
Trauma induces intestinal microbiota disruption in mice. Relative abundance of intestinal bacterial species, including *Lactobacillus*, *Lachnospiraceae NK4A136 group*, *Akkermansia*, *Mycoplasma*, *Dubosiella*, *Allobaculum*, *Bacteroides*, and *Parasutterella*, was assessed by 16S rRNA sequencing 14 days post-trauma, n = 7. *P < 0.05, **P< 0.01, ***P< 0.001, ns = no statistical significance vs. control.

To explore the impact of trauma on gut microbiota, the relative abundance of fecal microbiota at the genus level was analyzed using 16S rRNA sequencing 14 days post-trauma. Traumatized mice exhibited significant increases in the relative abundance of *Lactobacillus*, *Mycoplasma*, *Allobaculum*, and *Bacteroides* compared to controls (*P*<0.05). In contrast, *Lachnospiraceae NK4A136 group*, *Akkermansia*, and *Dubosiella* were significantly decreased (*P*<0.05), while Parasutterella remained unchanged (*P* > 0.05, **Figure 2**). Akk, a member of the genus *Akkermansia*, has been shown to restore gut microbiota balance and regulate brain function (32). However, whether AKK can modulate trauma-induced gut microbiota dysbiosis and cognitive impairments remains unclear.

### AKK treatment improves cognitive impairment and anxiety in traumatized mice

**Figure 3:**
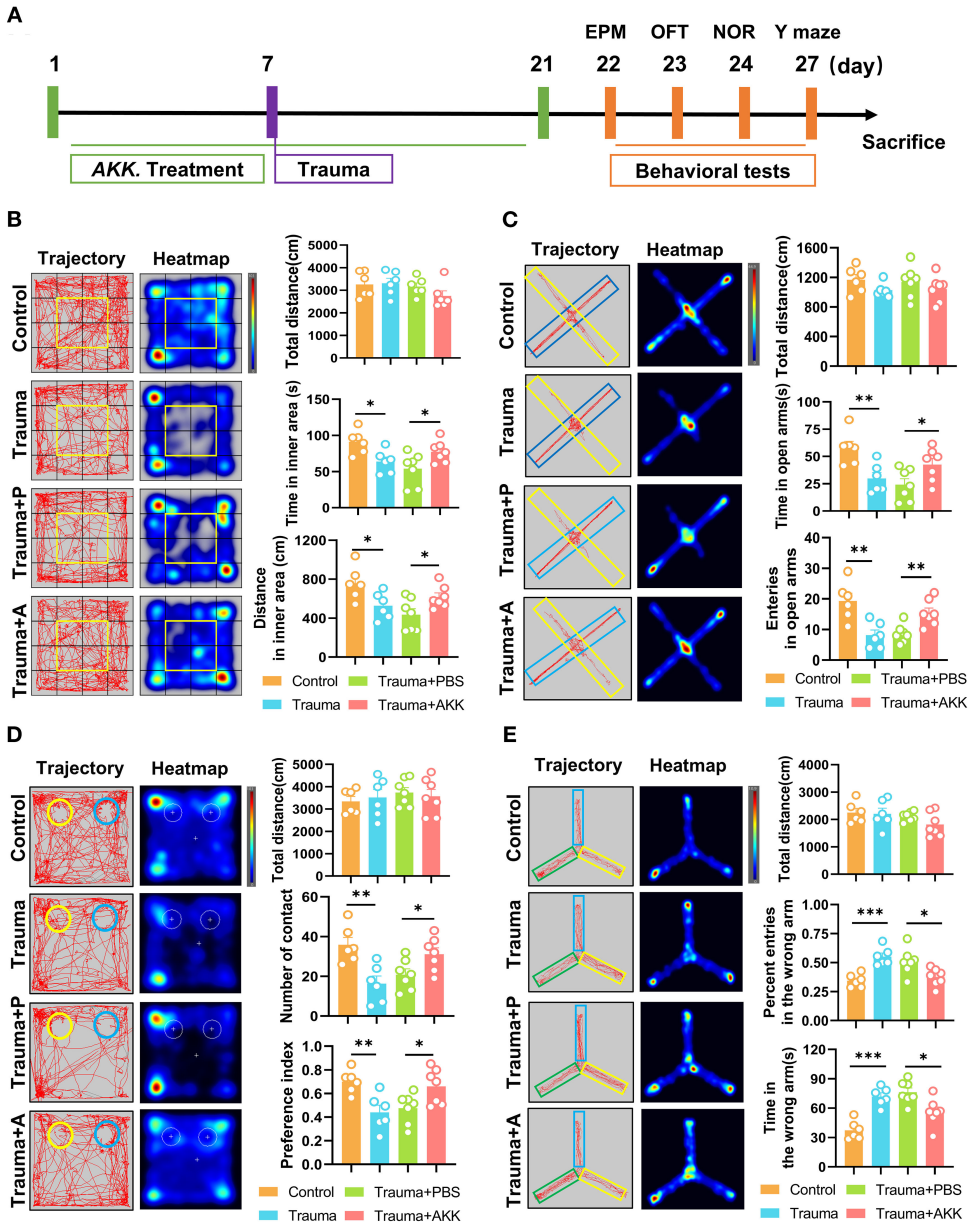
AKK treatment ameliorates cognitive dysfunction in trauma-exposed mice. **(A)** Behavioral testing timeline and treatment schedule for probiotic administration. **(B)** Behavior trajectory and heatmap from the OFT, divided into 16 sections, with the central 4 sections (highlighted in yellow) representing the inner area. The total distance (cm) in the open field, including the distance traveled in the inner area (cm) and time spent in the inner area (s), are also shown. **(C)** Behavior trajectory and heatmap from the EPM (yellow for the open arms, blue for the closed arms), with the total distance (cm) calculated. Entries into the open arms and time spent in the open arms (s) are also displayed. **(D)** Behavior trajectory and heatmap from the novel object recognition test (yellow for the novel object A2, blue for the familiar object A1), with the total distance (cm) calculated. The number of contacts with the objects and the preference index [T_A2_/(T_A1_+T_A2_)] are shown. **(E)** Behavior trajectory and heatmap from the Y-maze test (green for the food arm, yellow for the error arm, and blue for the start arm). The total distance (cm), percent entries in the wrong arm, and time in the wrong arm are also indicated. n ≥ 6, *P < 0.05, **P<0.01, ***P<0.001.

To investigate whether AKK treatment could alleviate behavioral abnormalities in traumatized mice, this study administered AKK and assessed behavioral changes (**Figure 3A**). In the OFT, AKK-treated traumatized mice (Trauma+AKK) showed no significant difference in total distance traveled compared to Trauma+PBS. However, they exhibited significantly increased distances and time spent in the inner area (*P*<0.05, **Figure 3B**). In the EPM test, traumatized mice had reduced exploration times and entries into the open arms compared to controls. AKK treatment significantly increased these parameters in traumatized mice (*P*<0.05, **Figure 3C**). In the NORT, AKK-treated traumatized mice showed no significant difference in total distance traveled, but had significantly increased exploration frequencies and preference indices for the novel object (*P*<0.05, **Figure 3D**). In the Y-maze test, the number of entries and the time spent in the wrong arm were significantly increased in the trauma-induced mice compared with the control group (*P <*0.001, **Figure 3E**). However, AKK treatment significantly reduced the number of entries and the time spent in the wrong arm in the trauma-induced mice compared with the Trauma + PBS group (*P <*0.05, **Figure 3E**). AKK treatment significantly improved cognitive function-related parameters. Overall, AKK treatment significantly improved anxiety-like behaviors and cognitive impairments in traumatized mice.

### AKK treatment restores gut microbiota dysbiosis

**Figure 4:**
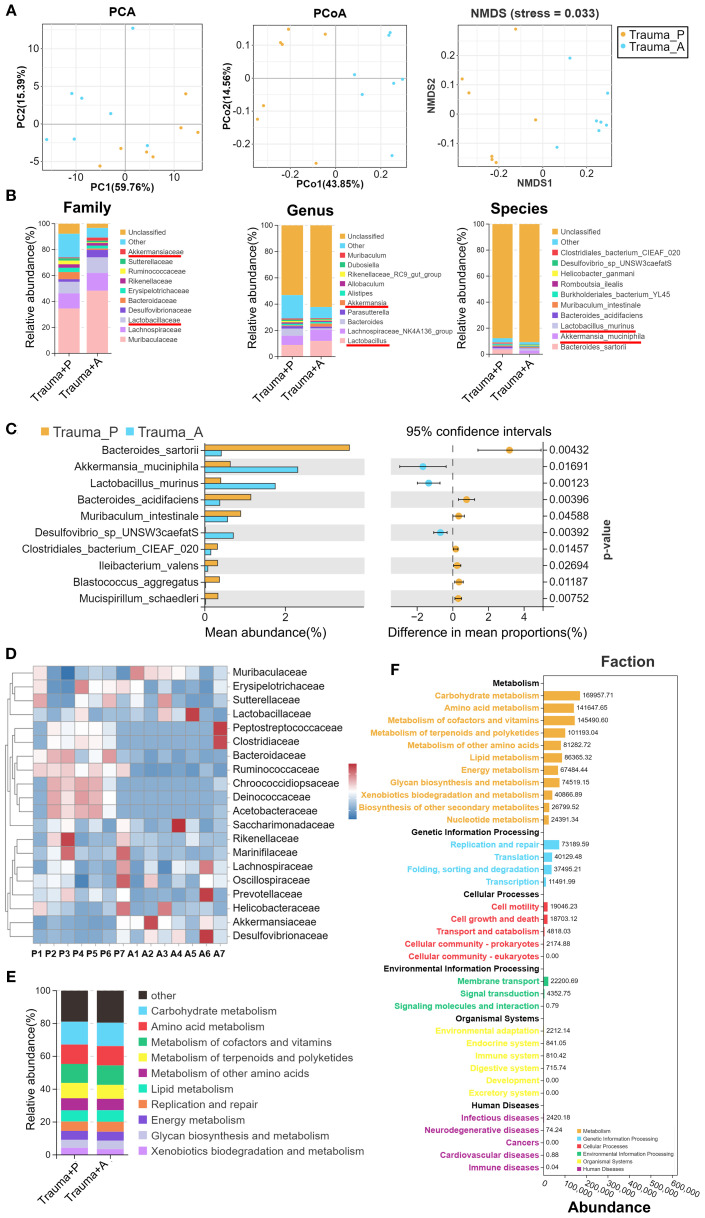
Gut microbiota 16S rRNA sequencing in traumatized mice. **(A)** β-diversity analysis of samples from the two groups, using PCA, PCoA, and NMDS. **(B)** Distribution stacked map for family, genus, and species. **(C)** Comparison of genus-level differences between the two groups, with Welch’s t-tests performed on the top 10 species showing statistical significance. **(D)** Family-level distribution heatmap, with “P” representing Trauma+PBS and “A” representing Trauma+AKK. **(E)** Stacked diagram of functional categories at Level 2 of the KEGG database. **(F)** PICRUSt2 Functional Distribution General Map. “Trauma P” represents Trauma+PBS, and “Trauma A” represents Trauma+AKK.

Growing evidence indicates that gut microbiota influences brain function and cognitive performance through the gut-brain axis, primarily *via* metabolites and neurotransmitters (33). Dysbiosis can disrupt intestinal permeability, allowing bacteria and toxins to enter the bloodstream, thereby inducing inflammation that impairs cognitive and emotional functions. To explore whether AKK treatment modulates trauma-induced changes in gut microbiota, 16S rDNA sequencing was performed to assess microbial composition and abundance in traumatized mice. Fourteen samples from two groups (n = 7 per group) were sequenced to generate V3-V4 16S rRNA gene profiles. Beta-diversity was evaluated using PCA, PCoA, and NMDS to determine microbial community similarity. PCA revealed distinct group separation (**Figure 4A**, left), with principal components PC1 and PC2 accounting for 59.76% and 15.39% of the variation, respectively. PCoA showed that PC1 and PC2 explained 42.85% and 15.56% of the variation (**Figure 4A**, middle). NMDS analysis, with stress = 0.033 (stress < 0.05), confirmed significant differences between samples (**Figure 4A**, right). AKK treatment was found to significantly alter the gut microbiota composition, with increases in *Lactobacillus* and *Akkermansia* at the family, genus, and species levels, while *Bacteroides* decreased (**Figure 4B**). To pinpoint bacterial taxa linked to AKK treatment in traumatized mice, Welch’s t-tests identified the top 10 core taxa explaining group differences. As shown in **Figure 4C**, AKK-treated traumatized mice (vs. Trauma+PBS) exhibited lower levels of *Bacteroides sartorii* (*P*=0.004), *Bacteroides acidifaciens* (*P*=0.004), and *Muribaculum intestinale* (*P*=0.046), while *Akkermansia muciniphila* (*P*=0.017) and *Lactobacillus murinus* (*P*=0.001) increased significantly. A heatmap of the top 20 species at the family level is provided in **Figure 4D**. Functional abundance heatmaps indicated associations with carbohydrate metabolism, amino acid metabolism, cofactor and vitamin metabolism, terpenoid and polyketone metabolism, other amino acid metabolism, and lipid metabolism (**Figure 4E**). Further functional predictions using PICRUSt2 revealed that trauma altered various biological processes in gut microbiota, particularly metabolism, genetic information processing, and cellular processes.

### AKK treatment modulates metabolite composition in traumatized mice

**Figure 5:**
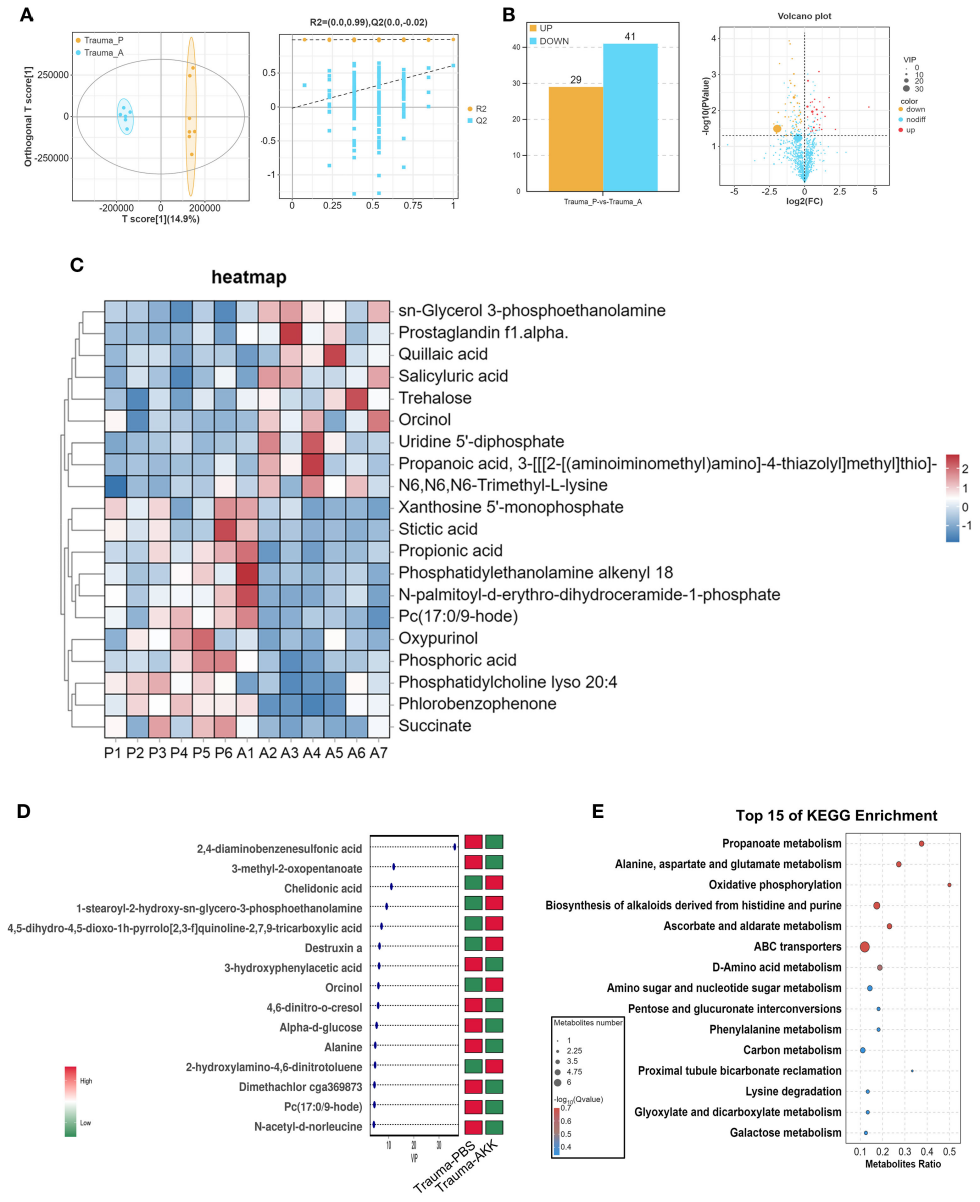
Serum metabolite sequencing in traumatized mice. **(A)** OPLS-DA score plot and permutation test plot comparing the groups. **(B)** Bar chart and volcano plot of differential gene expression. **(C)** Cluster heatmap of differential metabolite expression between the two groups, with “*P*” indicating Trauma+PBS and “A” representing Trauma+AKK. **(D)** Variable Importance in Projection (VIP) plot for differential metabolite expression between the two groups. **(E)** KEGG functional enrichment analysis of differentially expressed metabolites.

Given the ability of gut microbiota to influence the host through metabolites, metabolomics analysis was conducted on traumatized mice. OPLS-DA modeling (T score = 14.9%) and permutation tests confirmed the model’s reliability and predictability (**Figure 5A**). Bar and volcano plots displayed upregulated and downregulated metabolites based on statistical thresholds (*P*<0.05, |log2FC| > 1, **Figure 5B**). AKK treatment in traumatized mice (vs. Trauma+PBS) revealed 70 differential metabolites, with 29 upregulated and 41 downregulated. A heatmap illustrating the top 20 most differentially expressed metabolites between groups was also constructed (**Figure 5C**). Variable Importance in Projection (VIP) analysis identified key metabolites, such as a significant decrease in 2,4-diaminobenzenesulfonic acid and 3-methyl-2-oxopentanoate, and an increase in chelidonic acid, 1-stearoyl-2-hydroxy-sn-glycero-3-phosphoethanolamine, and 4,5-dihydro-4,5-dioxo-1h-pyrrolo[2,3-f]quinoline-2,7,9-tricarboxylic acid in AKK-treated traumatized mice (**Figure 5D**). KEGG enrichment analysis indicated that these differential metabolites were involved in alanine metabolism, alanine, aspartate, and glutamate metabolism, oxidative phosphorylation, histidine biosynthesis, purine-derived alkaloid biosynthesis, ABC transporters, and other biological processes (**Figure 5E**).

### Correlation analysis between metabolites and gut microbiota

**Figure 6:**
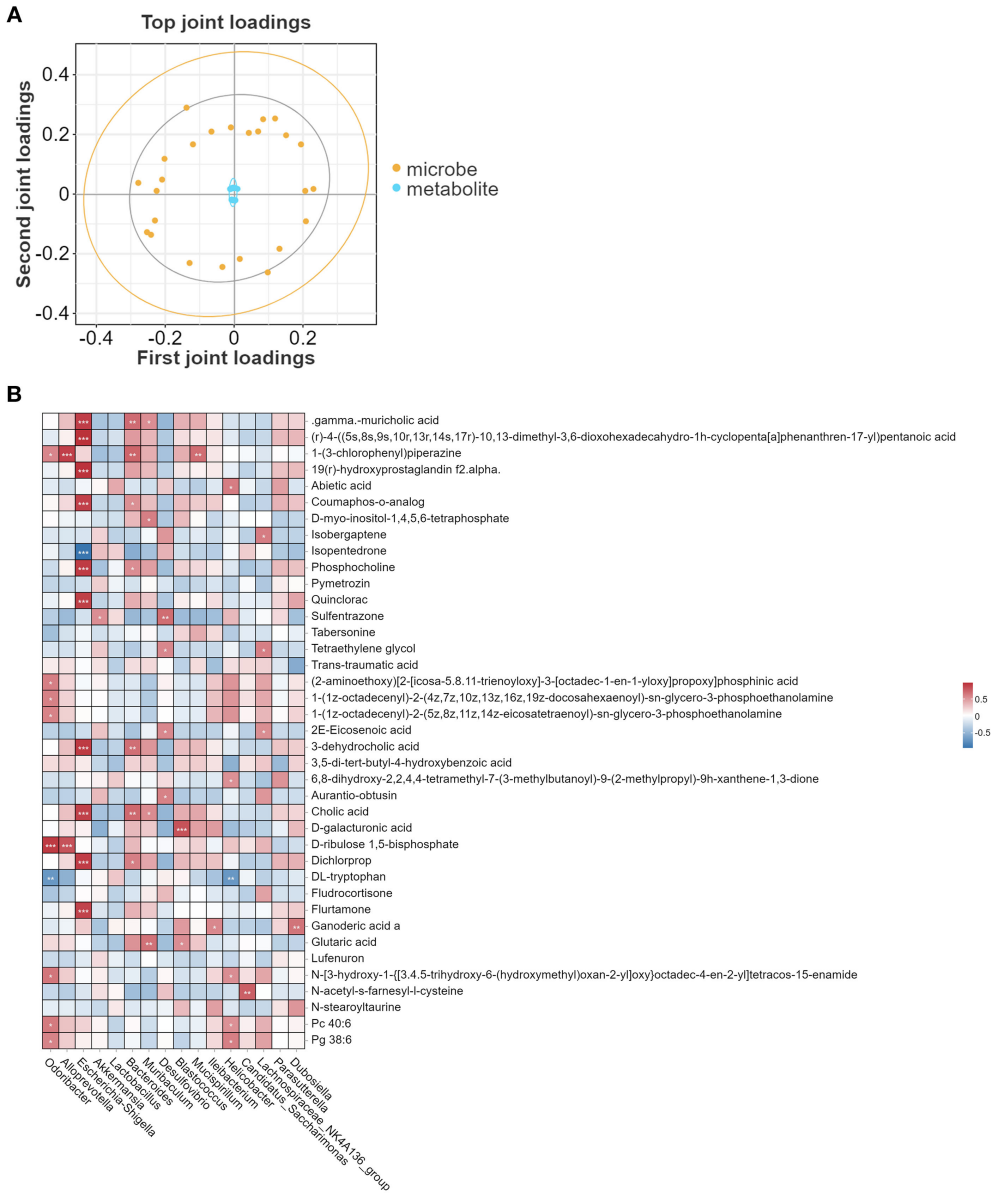
Correlation analysis between metabolites and gut microbiota. **(A)** O2PLS model illustrating the association between species and metabolites at the genus level. **(B)** Pearson correlation heatmap of metabolites and gut microbiota at the genus level. *P < 0.05, **P<0.01, ***P<0.001.

Subsequently, the correlation between gut microbiota and metabolites was explored. The O2PLS model loading plot (**Figure 6A**) demonstrated a strong association between the microbiota and metabolites. Further genus-level heatmap analysis revealed specific correlations between microbiota and metabolites. For example, *Escherichia-Shigella* exhibited a positive correlation with gamma- muricholic acid, 19(r)-hydroxyprostaglandin, coumaphos-o-analog, phosphocholine, and quinclorac, but a negative correlation with isopentedrone. *Odoribacter* showed a positive correlation with D-ribulose 1,5-bisphosphate but a negative correlation with DL-tryptophan (**Figure 6B**).

### AKK treatment alleviates neuroinflammation in traumatized mice

**Figure 7:**
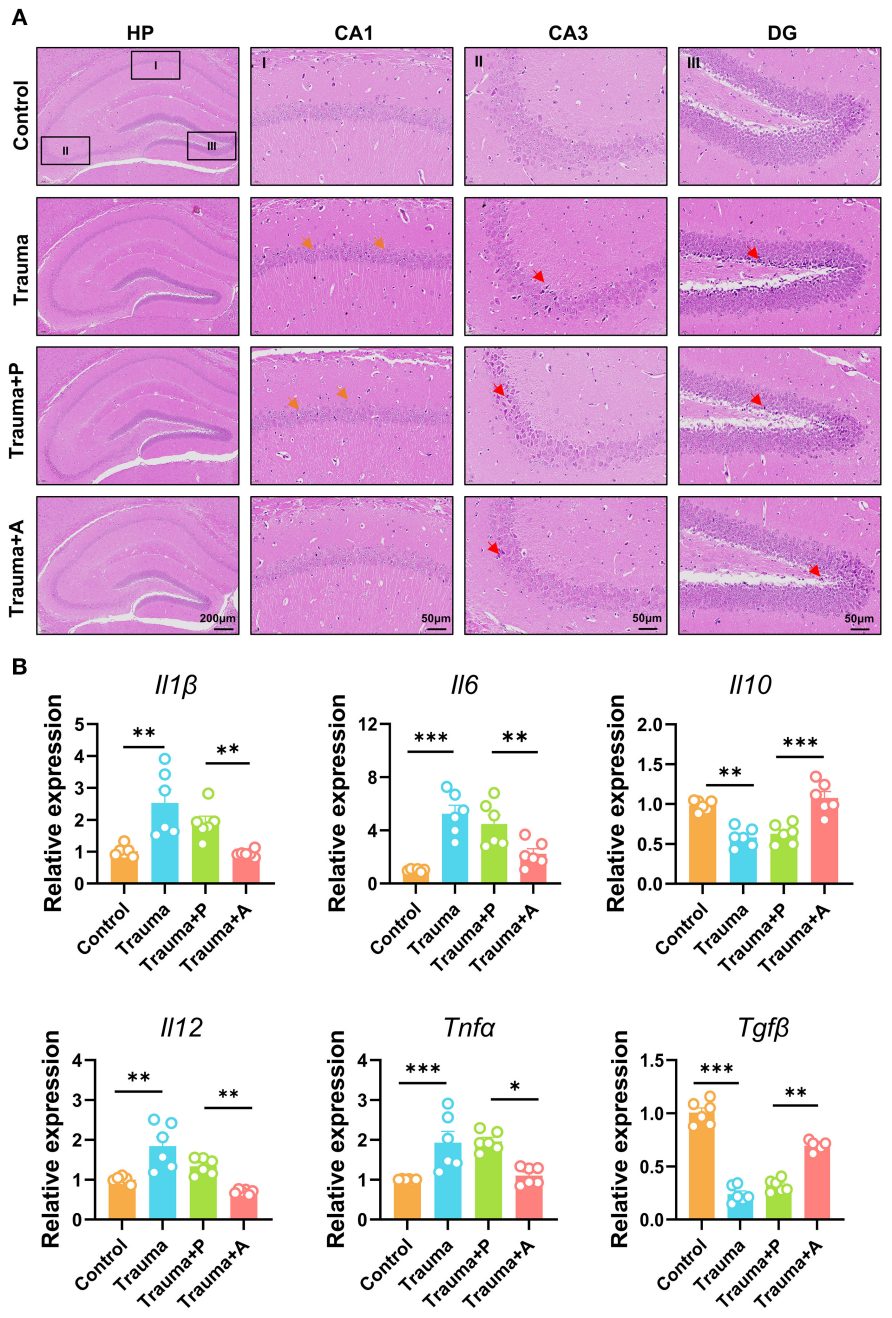
AKK treatment improves nerve damage and neuroinflammation in trauma-exposed mice. **(A)** HE staining of the hippocampus, with orange arrows indicating neuronal disturbances and red arrows showing alterations. HP, hippocampus; CA1, cornu ammonis 1; CA3, cornu ammonis 3; DG, dentate gyrus. HP scale: 200 µm; DG, CA1, CA3 scale: 50 µm. **(B)** Relative expression levels of cytokines (*Il1β*, *Il6*, *Il10*, *Il12*, *Tnfα*, and *Tgfβ*) and TNF-α mRNA in the mouse hippocampus, n = 6, **P<0.01, ***P<0.001.

To evaluate the neuroprotective effects of AKK, neuroinflammation in the hippocampus of traumatized mice was assessed through HE staining and cytokine expression analysis. The hippocampus, a key region in the limbic system, comprises the Cornu Ammonis (CA) 1, CA2, CA3, CA4, and Dentate Gyrus (DG), all of which are integral to memory formation, emotional regulation, and cognitive function. HE staining revealed that traumatized mice (vs. Control) displayed sparsely and disordered pyramidal neurons arrangement in the CA1 region, along with neuronal nuclear condensation and necrosis in the CA3 and DG regions. AKK treatment, however, significantly alleviated these pathological alterations in the hippocampus (**Figure 7A**). QPCR analysis indicated significantly increased levels of pro-inflammatory cytokines (*Il1β*, *Il6*, *Il12*, *Tnfα*) and decreased expression of the anti-inflammatory cytokine *Il10* and *Tgfβ* in the hippocampus of traumatized mice (*P*<0.05, **Figure 7B**). AKK treatment significantly attenuated the release of neuroinflammatory cytokines. In summary, AKK treatment substantially mitigated neuronal damage and neuroinflammation in traumatized mice.

### AKK treatment inhibits microglial activation and enhances the BDNF/TrkB signaling pathway

**Figure 8:**
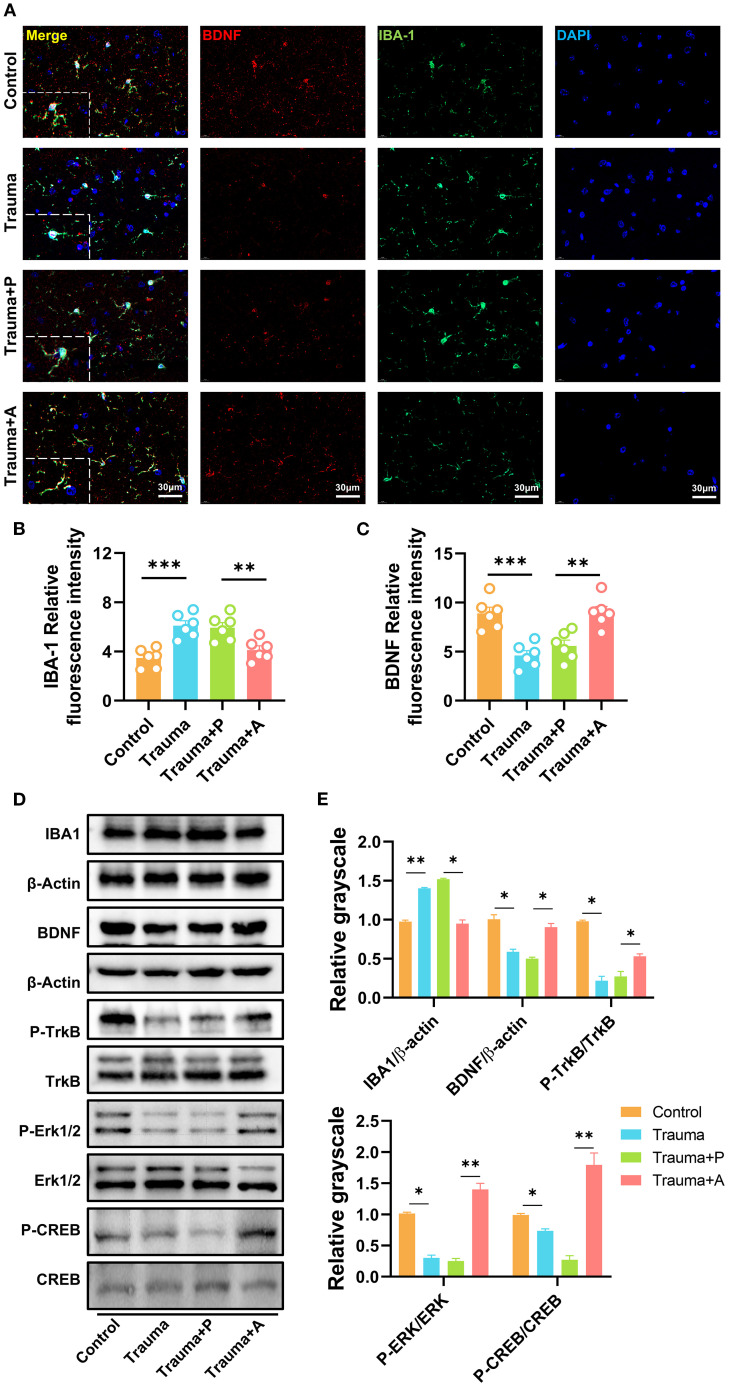
AKK treatment inhibits microglial activation and enhances the BDNF/TrkB signaling pathway. **(A)** Immunofluorescence staining of IBA1 and BDNF in the mouse hippocampus, with red indicating BDNF, green indicating IBA1, and blue indicating DAPI. Scale = 30 µm. **(B)** Quantification of IBA1 immunofluorescence, n = 6. **(C)** Quantification of BDNF immunofluorescence, n = 6. **(D)** Western blot analysis of hippocampal proteins. **(E)** Statistical analysis of Western blot results, n = 3, *P<0.05, **P<0.01.

Microbiota-derived metabolites, including SCFAs and neurotransmitters (e.g., neurotrophic factors, serotonin), can modulate neuroinflammation and microglial function by influencing blood-brain barrier permeability. To investigate the mechanisms by which AKK ameliorates neuroinflammation and cognitive impairments in traumatized mice, immunofluorescence staining for microglial marker IBA1 and BDNF was performed in the hippocampus (**Figure 8A**). In control mice, microglia exhibited a highly branched structure with three to four branches. Conversely, traumatized mice displayed enlarged microglial cell bodies with shorter and fewer branches. Quantitative analysis of IBA1 fluorescence intensity revealed a significant increase in IBA1-positive staining in the hippocampus of traumatized mice, indicating microglial activation, which was notably reduced following AKK treatment (**Figure 8B**). Additionally, BDNF fluorescence intensity was decreased in traumatized mice, an effect reversed by AKK treatment (**Figure 8C**). Western blot analysis further demonstrated that AKK treatment significantly reduced IBA1 expression in traumatized mice (**Figure 8D**). Furthermore, traumatized mice exhibited significant reductions in BDNF and phosphorylated Tropomyosin Receptor Kinase B (pTrkB) protein levels (*P*<0.05). Additionally, the levels of their downstream molecules pCREB and pERK were also significantly decreased (*P*<0.01). These reductions were restored by AKK treatment, while total TrkB, ERK, and CREB proteins remained unchanged (*P* > 0.05, **Figure 8E**). These findings suggest that AKK treatment inhibits microglial overactivation and promotes the BDNF/TrkB signaling pathway in the hippocampus of traumatized mice.

## Discussion

**Figure 9:**
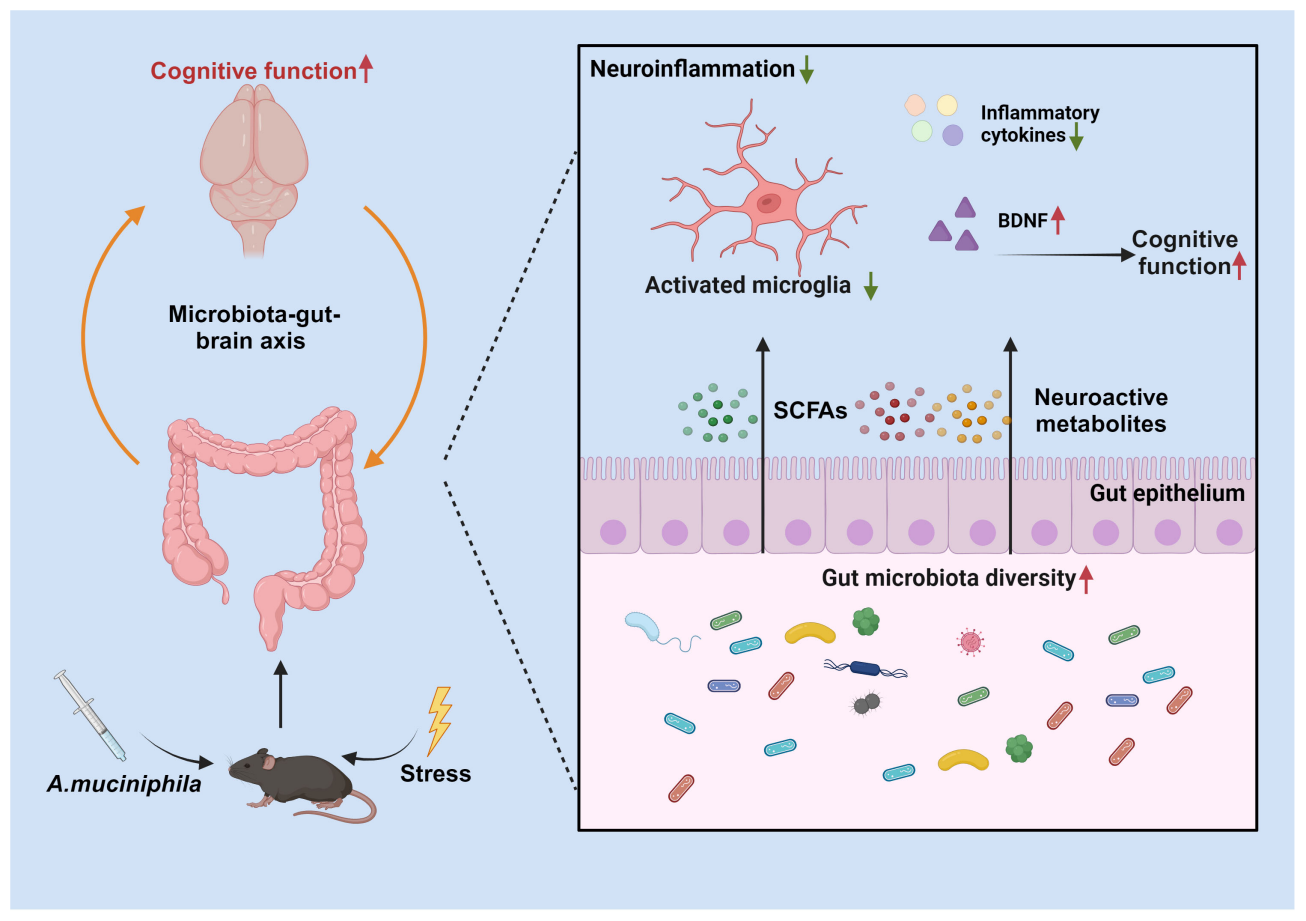
Hypothetical Mechanism of Trauma-Induced Cognitive Dysfunction Modulation by AKK in Mice. Supplementation with Akkermansia muciniphila (AKK) bacteria ameliorates trauma-induced cognitive dysfunction in mice *via* the microbiota-gut-brain axis. Specifically, AKK treatment enhances intestinal microbial homeostasis and metabolite production in traumatized mice. Through the production of short-chain fatty acids (SCFAs) and neuroactive substances, AKK inhibits the overactivation of microglia in the hippocampus. Furthermore, AKK improves cognitive function in mice by activating the BDNF/TrkB signaling pathway.

This study examined the therapeutic effects of AKK on cognitive impairments and neuroinflammation in a mouse model of blunt chest trauma. The results demonstrate that AKK treatment significantly ameliorated cognitive deficits and anxiety-like behaviors, while also reducing neuroinflammation in the hippocampus (**Figure 9**).

Blunt chest trauma is a common consequence of motor vehicle accidents and falls from heights, often leading to severe pain and respiratory distress, which can result in systemic hemodynamic instability, including hypotension and shock. These conditions can further exacerbate cerebral ischemia and hypoxia, contributing to cognitive impairments. During the acute phase, patients with blunt chest trauma often experience restricted mobility, decreased sleep quality, poor appetite, and heightened psychological stress, all of which can negatively affect cognitive function, manifesting as memory decline, inattention, and executive dysfunction (34). While most patients show some recovery in cognitive function after the acute phase, a subset may continue to experience long-term cognitive deficits (35). Our investigation revealed that employing a 300mm drop height in conjunction with a 50g weight successfully established a blunt chest trauma mouse model, which exhibited a 69% survival rate. The histological alterations detected in the pulmonary and cardiac tissues of the experimental mice corroborate the model’s capacity to emulate the quintessential pathological hallmarks of blunt chest trauma, including pulmonary hemorrhage, pulmonary edema, and myocardial injury. These findings are in alignment with prior research, which demonstrated that utilizing a 44-mm drop height along with weights of 138g, 190g, and 241g could generate an impact energy ranging from 0.13 to 0.23 J. This setup resulted in indentation depths of 12.4 to 14.7mm and rebound amplitudes of 0.6 to 1mm in mouse cadavers, without inducing rib fractures or dislocations, as evidenced by representative X-rays. Conversely, augmenting the drop height and weight can precipitate substantial intrathoracic hemorrhage, perforation or rupture of the right atrium, and lacerations in the lung lobes, frequently culminating in immediate mortality (26). The transient respiratory depression experienced by the surviving mice, followed by a gradual recovery of behavioral activity, indicates that this model is well-suited for examining the temporal dynamics of physiological and behavioral responses to trauma.

Behavioral tests in this study revealed that mice with blunt chest trauma exhibited significant reductions in exploration times and distances in the inner area of the OFT and decreased exploration frequencies and preference indices for novel objects in the NORT starting from day 14 post-trauma. These findings align with previous studies demonstrating that blunt chest trauma induces cognitive and emotional impairments in mice (36). The post-traumatic response is complex and may involve alterations in multiple neurotransmitter systems and neural circuits. Our findings suggest that the trauma model may primarily activate anxiety-related neural circuits, leading to the observed anxiety-like behaviors in the OFT and EPM. In contrast, the lack of significant depressive phenotypes in the TST and FST may indicate that the trauma model has a relatively minor impact on the neural circuits associated with depression. This differential impact on neural circuits could explain the observed phenotypic dissociation (37). Anxiety and depression, although often co-occurring clinically, involve distinct neural circuits. Anxiety is primarily associated with functional abnormalities in brain regions such as the amygdala, prefrontal cortex, and hippocampus, while depression is more closely linked to dysfunctions in the prefrontal cortex, nucleus accumbens, and raphe nuclei (38). Therefore, trauma may differentially impact these distinct neural circuits, leading to the observed phenotypic dissociation between anxiety and depression. This dissociation suggests that the trauma model may predominantly activate neural circuits associated with anxiety, while having a lesser impact on those related to depression. Furthermore, the hemodynamic alterations, neuroreflex responses, and inflammatory processes triggered by blunt chest trauma can lead to gastrointestinal dysfunction (39). Our analysis of gut microbiota composition revealed significant changes in the relative abundance of Lactobacillus, Bacteroides, and Akkermansia in traumatized mice 14 days post-injury, indicating trauma-induced dysbiosis. Previous research has shown that AKK can modulate gut microbiota and improve cognitive function through the alteration of SCFAs (32). Thus, whether AKK supplementation could alleviate cognitive and emotional impairments resulting from blunt chest trauma was further investigated.

In this study, AKK treatment significantly improved cognitive performance and reduced anxiety-like behaviors. Specifically, AKK-treated mice exhibited increased exploration times and frequencies in the NORT and Y-maze tests, indicating enhanced learning and memory abilities. Moreover, increased exploration interest in the OFT and EPM suggested reduced anxiety. These results align with the findings of Maftoon et al. (20). Numerous studies have demonstrated that AKK modulates the microbiota-gut-brain axis and plays a pivotal role in various neurological disorders. AKK regulates gut microbiota composition and metabolites, restores gut mucosal barrier integrity, modulates host immunity and neuroinflammation, and participates in the pathogenesis of neurological diseases (40). The underlying mechanisms likely involve AKK-derived SCFAs and other metabolites that influence neuroinflammation and neuronal health (41). 16S rRNA sequencing revealed that AKK treatment altered gut microbiota composition, significantly increasing the abundance of beneficial taxa such as *Lactobacillus* and *Akkermansia*. Non-targeted metabolomics analysis identified 70 differential metabolites in traumatized mice following AKK treatment, which were associated with alanine metabolism, alanine, aspartate, and glutamate metabolism, oxidative phosphorylation, biosynthesis of histidine and purine-derived alkaloids, and other biological processes. Previous studies have established a link between gut microbiota and cognitive impairments (42). For instance, the AKK-derived outer membrane protein Amuc_1100 can regulate L-arginine metabolism and mitigate age-related cognitive decline (43). AKK-derived exosomes have been shown to modulate serotonin metabolism in both the gut and hippocampus, preserving gut barrier and blood-brain barrier integrity, inhibiting inflammatory responses, and attenuating microglial TLR2/4 signaling, thereby improving postoperative cognitive dysfunction (44). *Lactobacillus* treatment has been reported to alleviate oxidative stress and inflammatory responses in high-fat diet-induced rats, improving cognitive function and mitigating neurodegenerative diseases (45). Furthermore, supplementation with *Lactobacillus rhamnosus GG* has been shown to increase synaptic proteins such as SYN and PSD-95 and BDNF in chronic ethanol-exposed mice, improving neuroinflammation and memory function (46). These changes in microbiota composition likely contribute to the observed improvements in cognitive and emotional outcomes, underscoring the critical role of gut microbiota homeostasis in maintaining metabolic and immune balance.

Microbiota-regulated metabolites and neurotransmitters can influence CNS inflammatory responses through the gut-brain axis (11). Neuroinflammation is a key factor in the pathophysiology of cognitive impairments following blunt chest trauma, where chronic inflammation exacerbates learning and memory deficits by affecting neurotransmitters and neural circuits (47). Elevated inflammatory markers in the serum, prefrontal cortex, and hippocampus of traumatized patients have been shown to impair cognitive function and emotional regulation (48). Our study demonstrated significant neuronal damage and increased inflammatory cytokine expression in the hippocampus of traumatized mice, consistent with previous findings (20, 22). AKK treatment significantly reduced pro-inflammatory cytokines such as IL-1β, IL-6, and TNF-α while increasing anti-inflammatory markers like IL-10, indicating its neuroprotective effects through modulation of inflammatory responses, in line with findings by Qiao et al. (49). Microglia, the resident immune cells of the CNS, play a pivotal role in the development and progression of neuroinflammation. Upon detecting pathological signals, microglia become activated and release pro-inflammatory cytokines such as IL-1β, IL-6, and TNF-α, which exacerbate neuroinflammation (50). Blunt chest trauma triggers the release of inflammatory mediators into the bloodstream, activating microglia in the brain (51). While activated microglia can clear pathogens and damaged cells, promote synaptic pruning and strengthening, and maintain brain homeostasis (52), excessive or chronic activation can lead to sustained inflammation, tissue damage, and neurodegeneration (50). Our results demonstrated that AKK treatment significantly reduced microglial activation in the hippocampus, consistent with previous reports (53). Additionally, microglia, astrocytes, and other cells secrete BDNF, a key regulator influencing neuronal survival, synaptic plasticity, and neuroinflammation (54). Immunofluorescence and western blot analyses revealed increased IBA1 (microglial) expression and decreased BDNF expression in the hippocampus of traumatized mice. Studies have shown that BDNF mRNA levels are downregulated in the hippocampus of rats following trauma (55). Plasma BDNF levels in traumatized patients are significantly lower than in healthy controls, impacting the BDNF/TrkB signaling pathway that regulates synaptic plasticity (56). Hauck et al. reported that BDNF expression initially increases shortly after trauma but decreases over time (57). Thus, microglial activation may be linked to the early increase in BDNF (58). BDNF, a key neurotrophic factor influencing cognitive function, has levels closely associated with cognitive abilities (59). BDNF binds to its high-affinity receptor TrkB, enhancing TrkB phosphorylation and activating downstream signaling pathways, such as CREB and ERK. These pathways promote neuronal survival and synaptic plasticity, thus regulating neuronal viability and influencing learning and memory functions (60, 61). Under traumatic stress, BDNF and its receptor TrkB expression vary depending on the duration and type of stress and the affected brain region, leading to neuronal degeneration and contributing to various CNS diseases (62).

Our study provides evidence that AKK treatment modulates gut microbiota and metabolites, which are associated with improvements in cognitive function and reductions in neuroinflammation following blunt chest trauma. However, there are several limitations to our study. First, the absence of fecal microbiota transplantation (FMT) experiments limits our ability to establish a causal relationship between microbial changes and neurobehavioral outcomes. Second, the lack of comparative analysis with established neuroprotective probiotics, such as Lactobacillus or Bifidobacterium species, restricts our understanding of AKK’s relative efficacy and mechanisms of action. Future studies should incorporate FMT and comparative analyses with well-known neuroprotective probiotics to provide a more comprehensive assessment of AKK’s therapeutic potential. Additionally, our microbiome and metabolomics analysis was conducted only at day 14 post-trauma, which does not fully capture the dynamic changes over the 60-day period during which behavioral recovery was observed. This temporal disconnect limits our ability to understand the relationship between gut microbiota changes and cognitive recovery patterns. Future studies should consider longitudinal sampling to elucidate the temporal dynamics of gut microbiota and metabolite changes in relation to cognitive recovery following blunt chest trauma (63, 64).

## Conclusions

In conclusion, our findings support the hypothesis that AKK could serve as a next-generation probiotic with the potential to alleviate cognitive impairments and neuroinflammation following trauma. This study emphasizes the critical role of gut microbiota in brain health and suggests that targeting the gut-brain axis may offer novel therapeutic strategies for managing trauma-related cognitive and emotional disturbances. Further research is needed to clarify the precise mechanisms by which AKK influences neuroinflammation and cognitive function and to explore its potential clinical applications in patients with trauma.

## Data Availability

The 16S rRNA data presented in the study are deposited in the NCBI BioProject repository, accession number PRJNA1227250.

## Glossary

AKK: Akkermansia muciniphila
BDNF: Brain-derived neurotrophic factor
CA: Cornu Ammonis
CNS: Central nervous system
DG: Dentate gyrus
EPM: Elevated plus maze
FST: Forced swimming test
HE: Hematoxylin-eosin
IBA1: Ionized calcium-binding adapter molecule 1
IF: Immunofluorescence
KEGG: Kyoto Encyclopedia of Genes and Genomes
NORT: Novel object recognition test
OFT: Open field test
PBS: Phosphate-buffered saline
PD: Parkinson’s disease
PE: Positive electrospray ionization
PI3K/mTOR: Phosphatidylinositol 3-kinase/mammalian target of rapamycin
qPCR: Quantitative real-time polymerase chain reaction
RDP: Ribosomal Database Project
SCFAs: Short-chain fatty acids
TrkB: Tropomyosin receptor kinase B
