# Supplementary material for: Akkermansia muciniphila alleviates cognitive impairment and neuroinflammation induced by blunt chest trauma
Source: Front Immunol. 2025 Oct 1;16:1657524. doi: 10.3389/fimmu.2025.1657524 (PMC12521121; doi:10.3389/fimmu.2025.1657524)
Supplement: Supplementary file 1 [file Supplementaryfile1.docx]

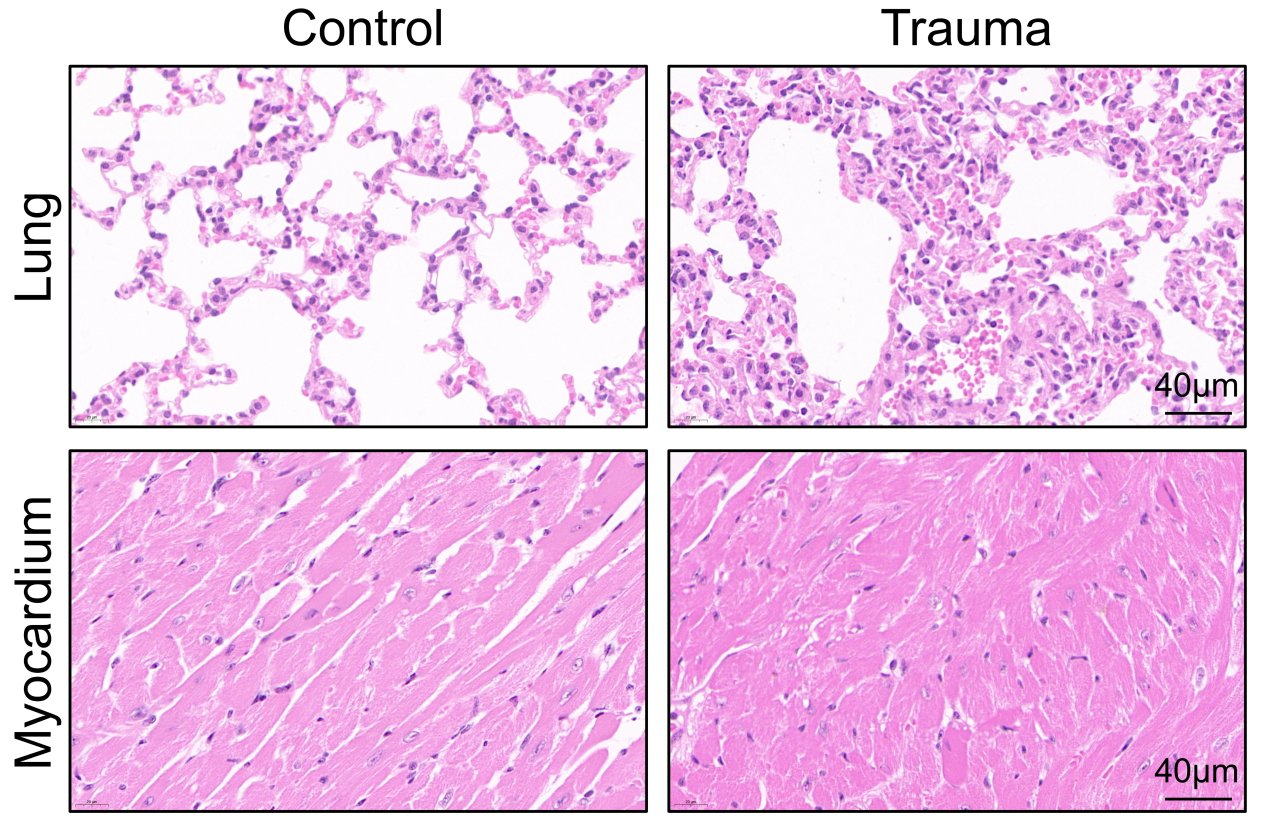


**Supplementary Figure 1. Trauma causes damage to the heart and lung tissues of mice.** H&E staining is used to observe the tissue structure of the heart and lungs in mice induced by trauma. Scale = 30 µm, n = 6.
