# Supplementary figures and images for: Akkermansia muciniphila alleviates cognitive impairment and neuroinflammation induced by blunt chest trauma
Source: Front Immunol. 2025 Oct 1;16:1657524. doi: 10.3389/fimmu.2025.1657524 (PMC12521121; doi:10.3389/fimmu.2025.1657524)

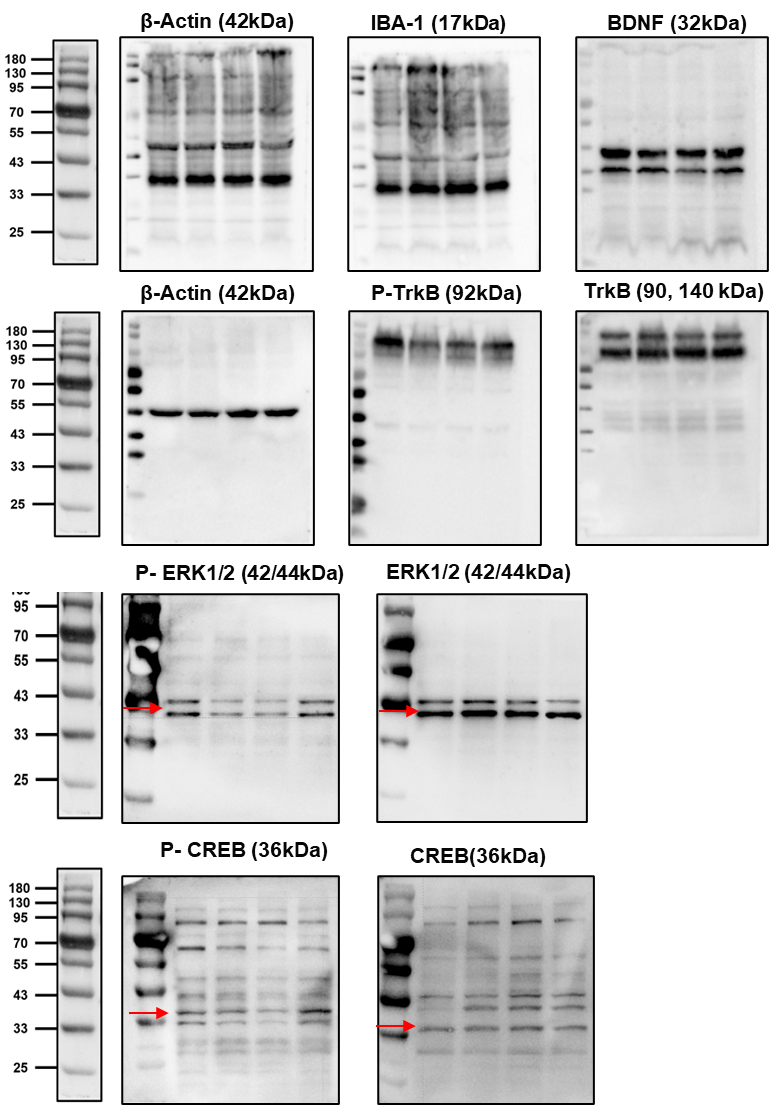


Supplementary Figure 2. Uncropped full Western blot membrane.

Supplement: Supplementary file 2 [file Supplementaryfile2.docx]
