# Supplementary material for: Akkermansia muciniphila alleviates cognitive impairment and neuroinflammation induced by blunt chest trauma
Source: Front Immunol. 2025 Oct 1;16:1657524. doi: 10.3389/fimmu.2025.1657524 (PMC12521121; doi:10.3389/fimmu.2025.1657524)
Supplement: Supplementary file 3 [file Table1.docx]

Supplementary Table 1 Primer for real-time PCR

| Gene | Sequence |
| --- | --- |
| IL-1β-F | 5'-TCCTGATGTTGCTGACCGT-3' |
| IL-1β-R | 5'-GATGCTGCTTCCAGGTTGTC-3' |
| IL-6-F | 5'-AGTGAGGAACAAGCCAGAGC-3' |
| IL-6-R | 5'-GCTGATCCACATCTGCTGGA-3' |
| IL-10-F | 5'-AGGGCACCCAGTCTGAGAACA-3' |
| IL-10-R | 5'-CGGCCTTGCTCTTGTTTTCAC-3' |
| IL-12-F | 5'-ATGAGGATGCTTCTGCATTTG-3' |
| IL-12-R | 5'-TCAACTTTCTATTATCCACTCGGTGTTCATTAC-3' |
| TNF-α-F | 5'-ATGAGCACTGAAAGCATGATC-3' |
| TNF-α-R | 5'-TCACAGGGCAATGATCCCAAAGTAGACCTGCCC-3' |
| TGF-β-F | 5'-AACATGATCGTGCGCTCTGCAAGTGCAGC-3' |
| TGF-β-R | 5'-AAGGAATAGTGCAGACAGGCAGGA-3' |
